# Frankly, My Error, I Don’t Give a Damn: Retrieval of Goal-Based but Not Coactivation-Based Bindings after Erroneous Responses

**DOI:** 10.5334/joc.224

**Published:** 2022-06-06

**Authors:** Juhi Parmar, Anna Foerster, Roland Pfister, Klaus Rothermund

**Affiliations:** 1 Friedrich-Schiller-Universität Jena, DE; 2 Julius-Maximilians-Universität Würzburg, DE

**Keywords:** action control, error processing, binding and retrieval, distractor-response binding, goal-based binding, coactivation-based binding

## Abstract

Previous studies demonstrated binding and retrieval of stimuli and correct responses even for those episodes in which the actual response was wrong (goal-based binding and retrieval). In the current study, we tested whether binding based on a co-activation of stimuli and erroneous responses occurred simultaneously with goal-based binding, which could have been masked by a more efficient retrieval of goal-based bindings in previous studies. In a pre-registered experiment (n = 62), we employed a sequential prime-probe design with a three-choice colour categorisation task. Including three different responses in the task allowed us to conduct separate tests for stimulus-based episodic retrieval of either the correct response (goal-based) or of the actual erroneous response (coactivation-based) after committing an error. Replicating previous findings, our study provides support for goal-based binding of stimuli and correct responses after errors, while showing that there is no independent coactivation-based binding of the erroneous response itself.

Human action control is highly adaptive in that it employs mechanisms that allow behaviour automatization to preserve cognitive resources whenever possible (Wood & Rünger, 2016). This adaptive system can create links between features of the stimuli in the environment (contexts, objects) and the actions that occur in close temporal contiguity with these events. Subsequent repetition of any such features (not only “target” features, but also task irrelevant features known as “distractors”; Rothermund et al, 2005) can then retrieve the previous binding, which leads to an automatic reactivation of the respective action (Frings et al., 2020). Geared towards behaviour automatization, the benefits of the systems underlying automatic response retrieval are evident in everyday life, with many instrumental activities becoming stimulus-dependent over the course of many repetitions. Known as stimulus response binding and retrieval (SRBR),^1^ this is important for the development of automatic control of behaviour (Frings et al., 2020; Hommel et al., 2001), and might also play a fundamental role in the formation of habits (Giesen et al., 2020).

Until now, experimental research on SRBR has more or less exclusively focused on correct action episodes, that is, responses that were conducted in accordance with the rules and instructions of the task. It has been proposed that SRBR only emerges for correct but not for erroneous actions (Hommel, 2005). Therefore, a pertinent question with regard to these binding and retrieval effects is whether, and how, they occur in case of errors, that is, for actions that were wrong, and were executed by mistake. Does one erroneous response lead to a perpetuation of the error via binding and retrieval, subsequently transforming a single mistake into a (bad) habit (Giesen et al., 2020)? Anecdotal evidence from daily life seems to support the notion of automatic repetitions of previous errors. For instance, playing a false note when practicing a piece of music on the piano can result in playing exactly the same false note at exactly the same position when playing the tune again the next time. This is not always the case, however, and in many situations, practicing a complex behaviour leads to continuous improvement despite initial errors, sometimes even due to error-based learning as in the case of language acquisition (Dell et al., 2019; Smalle et al., 2021).

In theoretical terms, a tendency to repeat an error if the previous situation is encountered again reflects the retrieval of the response that has actually been executed, recently introduced as retrieval of bindings based on co-activation (Foerster, Moeller, et al., 2021; Foerster, Rothermund, et al., 2021). Although in line with anecdotal experience from everyday situations, these previous studies did not produce supportive evidence for such a coactivation-based binding of stimuli and erroneous responses. Instead, task-relevant and task-irrelevant stimulus repetitions retrieved the to-be-executed response (i.e., the correct response), even after errors although this response was actually not carried out. These processes were labelled as goal-based binding, since they reflect a retrieval of the original intended or instructed rather than the actual response. A similar tendency to bind and retrieve the correct action was also found in a study investigating observational SRBR of actions performed by another actor that received negative feedback (Giesen et al., 2017).

In sum then, previous studies support the notion that relevant stimuli and irrelevant contexts are bound to what is perceived to be the most adequate response that is in line with the task rules or goals of an individual in that situation. Such goal-based binding and retrieval was observed even in cases where the desirable response was not executed, which suggests that intended action goals are stored in and retrieved from episodic bindings.

Crucially, however, previous studies tested binding and retrieval effects after errors in a way that the result pattern would either point to goal-based or to coactivation-based binding (Foerster, Moeller et al., 2021; Foerster, Rothermund, et al., 2021). With the possibility to orthogonally study effects of distractor repetitions without repeating the target (Rothermund et al., 2005), stimulus (here, distractor; see footnote 1) repetition effects were investigated in categorization tasks with only two response alternatives (Foerster, Rothermund, et al., 2021). For trials following action slips, goal-based binding is indicated by stimulus repetition benefits when the correct response repeats than changes across trials, whereas binding based on co-activation predicts exactly the opposite pattern (i.e., response time (RT) benefits for stimulus repetitions over stimulus changes in those sequences in which the required response changes relative to response repetition sequences.

As this form of testing does not allow for an independent assessment of both kinds of binding mechanisms, the results obtained in the previous studies only allow for a relative conclusion: Binding and retrieval based on co-activation should be considered the weaker or less frequently used process compared to goal-based binding. Due to an assessment of error-related retrieval effects that juxtaposes the two forms of binding, the lack of support for binding based on co-activation does not warrant the conclusion, however, that this mechanism does not exist. Instead, the more dominant process of goal-based binding might simply have masked binding by co-activation and thus prevented its detection. This speculation receives tentative support from the observation of consistently smaller binding and retrieval effects after errors than after correct responses (Foerster, Moeller, et al., 2021; Foerster, Rothermund, et al., 2021), and we therefore aimed at testing whether both types of binding and retrieval might indeed occur alongside each other.

## The present study

The present study investigated the possibility that goal-based and coactivation-based SRBR might both occur when processing an action slip. This was done by introducing a third response option in the study design. In a speeded-choice colour categorisation task in which three colours were mapped onto three response keys, participants were required to respond (under time pressure) to the colours (targets) of words (distractors, which they were asked to ignore). As in previous studies, we captured SRBR effects in a sequential prime-probe design by manipulating the relation of task irrelevant distractors (words) between prime and probe. This design allows for assessing stimulus repetition effects (SRE; performance in stimulus change trials – performance in stimulus repetition trials) for each response relation in the probe, indicating automatic retrieval of the response that was bound to the word during the prime episode (Rothermund et al., 2005; see also Frings et al. 2007). Facilitation (interference) effects indicate that a response was retrieved from the prime that matches (conflicts with) the response that is required in the probe.

**Figure 1:**
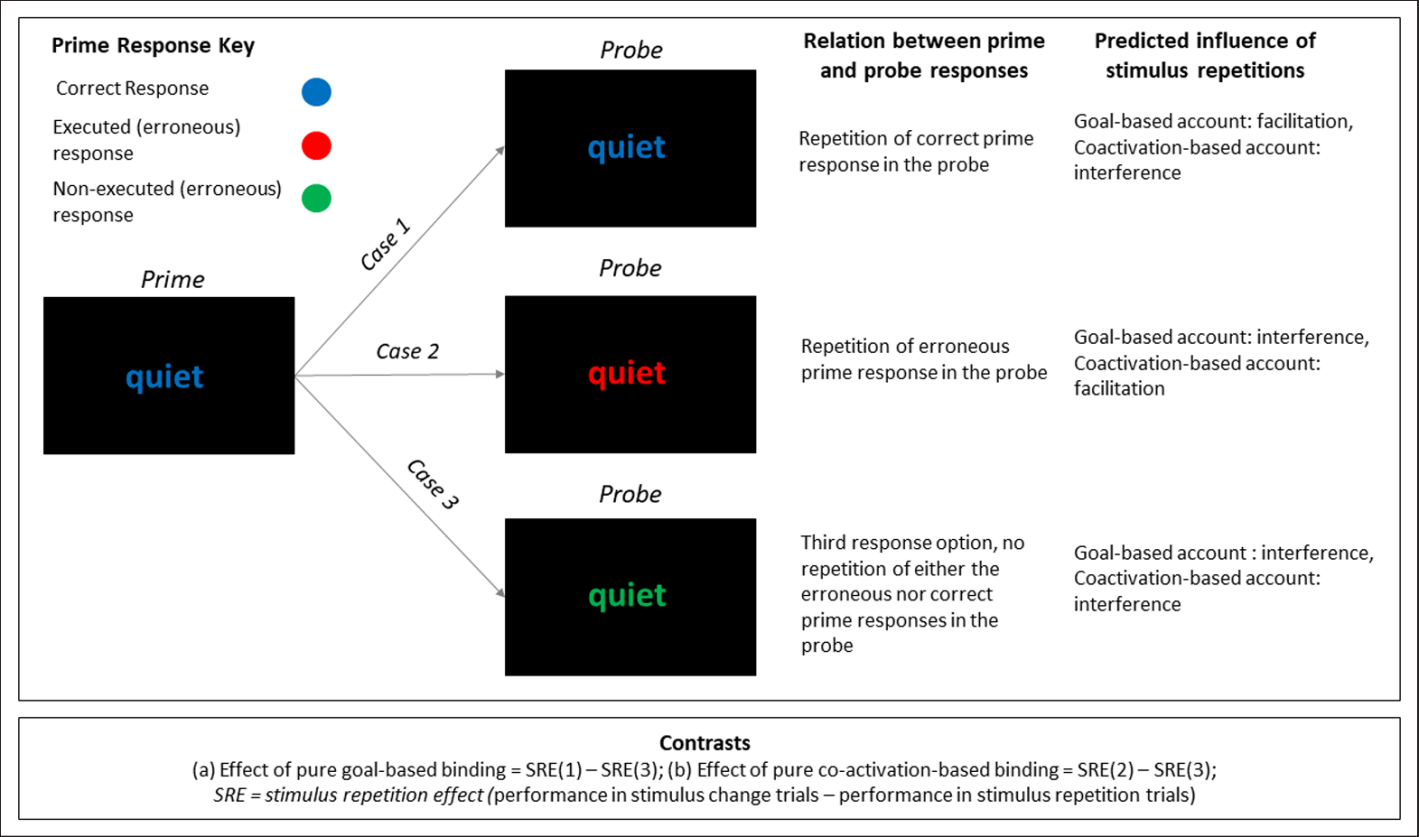
Possible prime-probe sequences after erroneous prime responses. *Note*: Case (1) Correct response repetitions: The correct response of the prime is also the correct response in the probe. Case (2) Error repetition: The actual (erroneous) response of the prime becomes the correct response in the probe. Case (3) Unrelated response sequence: The probe response corresponds neither to the actual (erroneous) prime response, nor to the correct prime response, but corresponds to the remaining third response. Predicted stimulus repetition effects (SRE; word change – word repetition) are listed in the right-most column: goal-based binding and retrieval predicts interference for response sequences (2) and (3), but facilitation for sequence type (1); coactivation-based binding and retrieval predicts facilitation for sequence type (2), and interference in sequences (1) and (3). The contrast of stimulus repetition effects for (3) and (1) is a pure indicator of goal-based binding and retrieval, the contrast of stimulus repetition effects for (2) and (3) is a pure indicator of coactivation-based binding and retrieval.

With three response keys, it is possible to disentangle patterns for goal-based and coactivation-based binding and retrieval after errors, providing independent tests for both types of retrieval. Our design contained three types of response sequences after an error occurred during the prime (see Figure 1): (1) The same response that was required (but not executed) during the prime was repeated as the required (correct) response during the probe (“repetition of the correct response”), (2) the same response that was executed (erroneously) during the prime became the required (correct) response during the probe (“repetition of the erroneous response”), and (3) the required response during the probe corresponded neither to the (erroneously) executed response during the prime, nor to the required (correct) response during the prime (“unrelated response option”).

The relation of task irrelevant distractors (words) and responses allowed us to disentangle influences of goal-based and coactivation-based binding and retrieval (see Figure 1). Specifically, for sequences in which the correct prime response was repeated as the correct response in the probe (case 1), repeating compared to changing the irrelevant stimulus should produce facilitation if there was retrieval of goal-based bindings, but should produce interference if there was retrieval of bindings based on co-activation. For sequences in which the erroneous response of the prime became the correct response in the probe (case 2), repeating the irrelevant stimulus should produce facilitation if there was retrieval of bindings based on co-activation, but should produce interference if there was retrieval of goal-based bindings. For sequences in which the third response option was the correct response in the probe (i.e., when neither the erroneous nor the correct prime response became the required response during the probe, case 3), repeating the irrelevant stimulus should produce interference due to the retrieval of both coactivation-based, as well goal-based bindings.

Contrasting SREs after prime errors for different types of response sequences yields pure indices of binding and retrieval that reflect either goal-based or coactivation-based binding (see Figure 1): Contrasting stimulus repetition effects for case (1) and (3) gives a pure index of goal-based binding and retrieval (costs of binding based on co-activation are identical for these sequences and cancel each other out in the contrast). Similarly, contrasting stimulus repetition effects for (2) and (3) gives a pure index of binding and retrieval that is based on co-activation (costs of goal-based binding are identical for these sequences and cancel each other out in the contrast).

## Method

### Sample and Pre-registration

The experiment was preregistered at osf.io/a6yuw before commencing data collection, and ethical approval was granted by the local ethics committee. A power analysis had suggested a sample size of 54 participants to yield a power of 95% to detect a moderate effect size (*d_z_* = 0.5) in a two-tailed paired-sampled *t*-test, with alpha-level at 5% according to an *a-priori* power-analysis using G*Power (Version 3.1.9.4; Faul et al. 2007). This sample size further ensured a power of 89% for detecting goal-based binding and retrieval effects as observed in Exp. 1 of Foerster, Moeller, et al. (2021; *d_z_* = 0.44). Considering unusable data and dropouts, we tested 62 participants (35 females; 27 males; *Mdn* age = 23 years). Data was collected from German-speaking students of Friedrich-Schiller University Jena through on-campus recruitment using posters and flyers. Participants were paid at the rate of Euro 8 per hour of their time, with an average experiment duration of 20–30 minutes.

### Design

For sequences with correct prime responses, the study has a 2 (distractor relation: word repetition vs. word change) × 2 (response relation: response repetition (same correct response in prime and probe] vs. response change [correct response changed from prime to probe]) within design with repeated measures on both factors. Standard SRBR effects are tested via the interaction of the two factors, with retrieval of the prime response being indicated by larger facilitation effects of word repetitions if the response repeats from prime to probe than for response changes (Rothermund et al., 2005).

For sequences with erroneous responses in the prime, our study has a 2 (distractor relation: word repetition vs. word change) × 3 (response relation: correct repetition [correct prime response repeated as correct response in the probe] vs. error repetition [erroneous prime response became correct probe response] vs. unrelated response [correct probe response was unrelated to either the erroneous or correct prime responses]) within design with repeated measures on both factors. Effects of error-based retrieval are tested by an interaction of the two factors. To separate retrieval of goal-based bindings and bindings based on co-activation, the response relation factor is split into two contrasts (c1: unrelated vs. correct repetition; c2: unrelated vs. error repetition). An interaction of distractor relation with the first contrast of the response relation factor is used to test pure effects of goal-based binding and retrieval, whereas the interaction of distractor repetition with the second contrast tests pure effects of binding and retrieval based on co-activation.

### Apparatus and stimuli

The lab was controlled for noise and light, with a capacity of 8 participants to simultaneously undertake experiments in partitioned cubicles. Stimuli were presented on a 16-inch monitor and participants responded on a standard German QWERTZ keyboard. The letters G, H, and J were marked with circular red, blue, and yellow coloured stickers, respectively, covering the letters on the keys, for the colour classification task. The target colour by response key combination was not counterbalanced for participants since no known association/heuristic between the colours and keys was predicted. However, in the event that such an association were to be formed, it would only result in a main effect of key press (evenly distributed across conditions), without any influence on SRBR effects, which are of main interest in this study. The entire experiment (including instructions in white letters and stimuli in task-relevant colours) was presented against a black background. All instructions, feedback, and stimuli were in German. Three neutral adjectives were presented as distractor words, namely “klein” (small), “leise” (quiet), and “weich” (soft), creating a total of nine possible stimuli (3 distractor words × 3 target colour combinations).

### Procedure

Before beginning the experiment, participants provided consent on paper via signature, as well as digitally, by pressing the key “J” on the keyboard after reading information about the experiment and its purposes. Participants were instructed to respond only to the colour of the word – the word itself was not important. They were informed about the response deadline (see next paragraph), and warned that although they should expect to commit errors, they must be as quick and accurate as possible.

Given that the aim of this experiment was to study errors, it was important to generate a sufficiently high error rate (aim: 25% errors) to provide enough trials for analysis, while at the same time maintaining meaningful responding above chance level. To address this issue, we introduced a response deadline to pressure participants into committing more errors. The response deadline was calculated and updated individually per participant after every block, by using their error rate from the previous block. For blocks where the preceding error rates were lower than 15%, 75 ms were subtracted from the response deadline for the next block, for error rates higher than 35%, 75 ms were added, and for the range in between (the desired error rate), the response deadline for the previous block was maintained.

A minimum response deadline was set at 275 ms. Following each block, if error rates were higher than 35%, participants received feedback to make fewer errors. In case of the other two error ranges, participants received information about the percentage of responses within the deadline, as well as an update that they should “keep it up”, or that the task would get faster. Lastly, participants with more than 25% responses after the response deadline were encouraged to respond accurately but faster. Following the deadline, the word turned to white, but stayed on the screen until the participant responded. To improve the chances of collecting less noisy data, participants were incentivised with receiving a chocolate bar at the end of the experiment if they made more than 65% accurate responses and ensure that more than 70% of their responses were within the response deadline.

Participants had to complete three practice blocks consisting of eighteen trials each, so they could become familiar with the task and the response deadline, for which a different set of distractor words were used. At least twelve (2/3) correct responses were required in each block, to proceed to the next, and for proceeding to the main experiment. Participants had to repeat the practice block until they achieved the required accuracy rate. The first block had no response deadline, the second practice block had a deadline of 750 ms, and the third had a deadline of 500 ms. Based on their error rates in the third block, participants would begin the first experiment block with a deadline of either 425 ms (500–75 ms due to an error rate <15%) or 500 ms (error rate between 15 and 35%). The respective colour-response mapping sequence remained on screen for the practice block, but not for the experimental blocks. No error feedback was provided during the experiment.

There were 23 experimental blocks, with 27 trials each, resulting in 621 trials in total (or 598 prime-probe pairs, i.e, trials 1–26 in each block qualified as primes for the immediately following trial, respectively, i.e., trials 2–27 qualify as probes) that could be used for the analysis of episodic SRBR effects. Specifically, for our analyses, this translates to approximately 150 trials with erroneous primes per participant, with 50 trials per error type, resulting in a minimum of 17 trials per condition of the 2 (distractor relation) × 3 (response relation) design per participant. This corresponds to previous studies that were conducted to investigate binding/retrieval effects (Frings & Moeller, 2010; Giesen et al., 2012; Giesen & Rothermund, 2014; Moeller et al., 2016). There were no colour-word contingencies, meaning each colour had an equal chance of appearing in each distractor word. Additionally, every distractor word and colour had an equal probability of being displayed.

**Figure 2:**
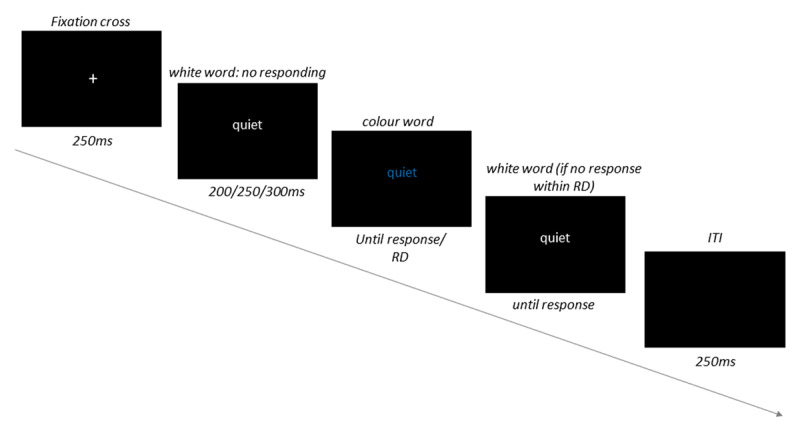
Trial procedure in experimental blocks. *Note*: Every trial started with the fixation cross, followed by the distractor word in white (during which period participants were not allowed to respond), the word then changed to one of the 3 target colours (here, the participant could respond). If the response deadline (RD) elapsed without a response, the word turned to white again, until response. Next, participants were shown a blank screen (ITI: inter-trial interval), followed by the next trial sequence.

Each trial started with the presentation of a fixation cross in white at the centre of the screen for 250 ms, followed by the distractor word in white lettering presented at random for 200 ms, 250 ms, or 300 ms for every trial (during which period participants would have been unable to respond), and then in a target colour (see Figure 2). If a response was not received before the response deadline elapsed, the word would turn white again, and stay on the screen until a response was received. After this, the inter-trial interval (a blank screen) lasted for 250 ms, followed by the fixation cross, and then the next trial sequence.

## Results

### Data pre-processing

**Figure 3:**
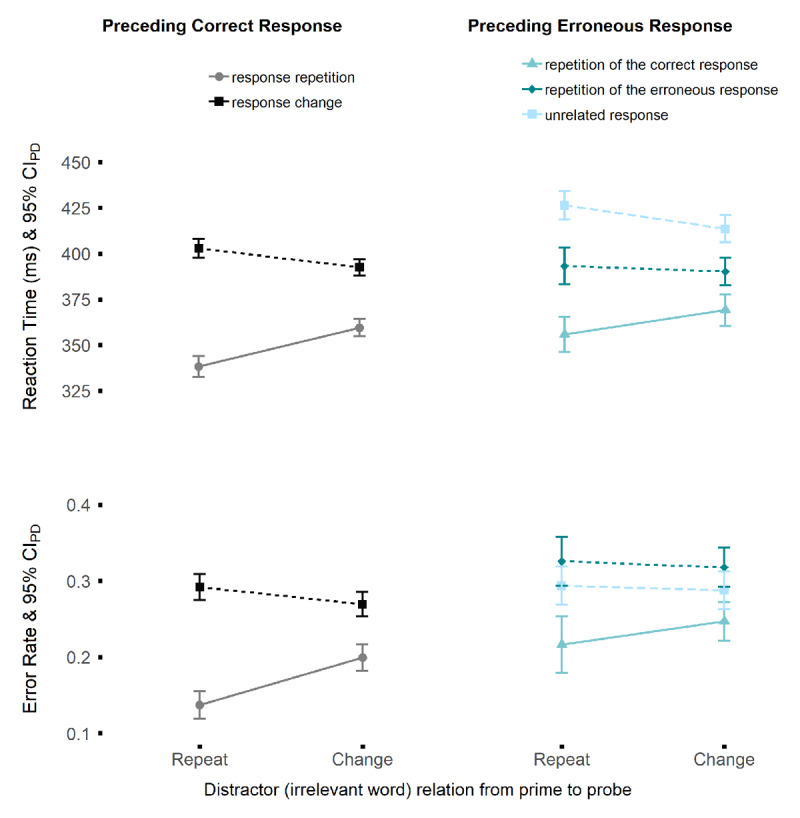
Overview of the results. *Note*: Mean response times (RTs; top) and error rates (bottom) for the analysis of binding and retrieval effects in probe trials after correct responses (left panels) and erroneous responses (right panels). Data are plotted as a function of distractor word relation (repetition vs. change) and response sequence (correct primes: response repetition vs. change; erroneous primes: repetition of the correct response, repetition of the erroneous response, unrelated response).

Our main analyses focused on post-error trials so that we selected trial sequences with an erroneous response (prime), followed by a correct response (probe) for RT analysis and sequences with an erroneous response (prime) followed by either another erroneous response or by a correct response (probe) for error analysis. This procedure naturally excluded the first trial of every block since these trials had no immediately preceding prime trial. Trials with RTs faster than 150 ms (1.5%) or slower than 1.5 times the interquartile range of the individual RT distribution (2.1%; outlier values according to Tukey, 1978) were excluded from the RT analyses. No participant was excluded from analyses owing to good quality data and high accuracy rates (minimum 65%). Thus, data is reported from a high-powered study, with a full sample size of 62 participants. Figure 3 provides an overview of the descriptive data for each design cell.

### Standard SRBR effects after correct prime responses

For sequences with correct prime responses, we found significant main effects for the factor distractor relation on RTs, *F*(1, 61) = 20.86, *p* < .001, η_p_^2^ = .26, and error rate alike,^2^ *F*(1, 61) = 11.61, *p* < .001, η_p_^2^ = .16. The same was true for the main effect of response relation for RTs, *F*(1, 61) = 160.51, *p* < .001, η_p_^2^ = .73, and error rate, *F*(1, 61) = 85.22, *p* < .001, η_p_^2^ = .58. This indicates that on average, following a correct response in the prime, probe responses were faster and more accurate for distractor repetition sequences (RT: *M* = 371, *SD* = 39; error rate: *M* = 0.21, *SD* = 0.05) compared to distractor change sequences (RT: *M* = 376, *SD* = 39; error rate: *M* = 0.23, *SD* = 0.06), as well as for response repetition sequences (RT: *M* = 349, *SD* = 36; error rate: *M* = 0.17, *SD* = 0.08) compared to response change sequences (RT: *M* = 398, *SD* = 46; error rate: *M* = 0.28, *SD* = 0.06).

Importantly, the two main effects were qualified by a significant two-way interaction for both RT, *F*(1, 61) = 90.86, *p* < .001, η_p_^2^ = .60, as well as error rate, *F*(1, 61) = 47.70, *p* < .001, η_p_^2^ = .44, attesting to standard SRBR effects. Distractor repetitions led to faster (*t*(61) = 9.06, *p* < .001, *d_z_* = 1.15) and more accurate (*t*(61) = 6.65, *p* < .001, *d_z_* = 0.85) probe responses in response repetition sequences, but led to interference in response change sequences (RT: *t*(61) = –6.18, *p* < .001, *d_z_* = –0.79; error rate: *t*(61) = –3.01, *p* = .004, *d_z_* = –0.38), indicating that in case of distractor repetitions, the prime response was retrieved from episodic memory, which matched the probe response in response repetition sequences, but led to a mismatch when the response changed from prime to probe.

### Retrieval effects after erroneous prime responses

For probes that were preceded by an erroneous prime trial (total 24.5% or 9,415 trials), the 2 (distractor relation: repetition vs. change) × 3 (response relation: repetition of the correct response vs. repetition of the erroneous response vs. unrelated response) ANOVA produced significant main effects for the factor response relation on RT, *F*(2, 60) = 58.70, *p* < .001, η_p_^2^ = .66, and error rates, *F*(2, 60) = 9.97, *p* < .001, η_p_^2^ = .25, but not for distractor relation (*F* < 1 for RT and error rate). On average, probe responses were fastest for sequences in which the correct prime response was repeated in the probe (although the correct prime response was not executed; *M* = 363, *SD* = 48), followed by sequences in which the prime error was to be repeated in the probe (*M* = 392, *SD* = 57), and by responses in the unrelated response condition (*M* = 420, *SD* = 50). Probe accuracies were highest (lowest error rates) in the correct response repetition condition (*M* = 0.23, *SD* = 0.10), followed by the unrelated response condition (*M* = 0.29, *SD* = 0.11), and were least accurate in the condition in which the erroneous prime response became the correct response in the probe (*M* = 0.32, *SD* = 0.13).

Most important for our research question, the two-way interaction was significant for RTs, *F*(2, 60) = 11.15, *p* < .001, η_p_^2^ = .27, but not for error rates, *F*(2, 60) = 1.37, *p* = .26 η_p_^2^ = .04. Following up on the significant interaction for the RT data, we split the interaction into planned contrasts (see Design section above), which revealed a significant interaction of distractor relation with the first contrast (unrelated vs. correct repetition) of the response relation factor *F*(1, 61) = 21.69, *p* < .001, indicating that distractor repetitions retrieved the correct response from the prime although this response had not been executed (retrieval of goal-based bindings). The part of the interaction that was due to the second contrast (unrelated vs. error repetition) was not significant, *F*(1, 61) = 2.46, p = .12, indicating that retrieval of bindings based on coactivation of stimuli and erroneous responses did not influence responding in the probe.

### Exploratory Analyses

Although not preregistered, on the recommendation of a reviewer, we additionally ran Bayes Factor analyses using JASP (v. 0.16.2.0) for the goal-based as well as coactivation-based retrieval contrasts, using scale parameter 0.71 (default) for the prior distribution. In the RT data, we found strong evidence for the goal-based retrieval hypothesis compared to the null hypothesis with BF_10_ = 1061.99, whereas for the coactivation-based retrieval hypothesis, we report anecdotal evidence in favour of the null hypothesis with BF_01_ = 2.26. In the error rates, we report anecdotal evidence in favour of the null hypothesis compared to the goal-based retrieval hypothesis with BF_01_ = 2.63. This was also the case for the coactivation-based retrieval hypothesis, where BF_01_ = 7.15 provides moderate evidence in favour of the null hypothesis.^3^

## Discussion

The present study explored the possibility of goal-based binding and coactivation-based binding both, occurring in the face of action slips. This was done against the background of previous work that had reported clear-cut evidence only for goal-based binding and retrieval (Foerster, Moeller, et al., 2021; Foerster, Rothermund, et al., 2021): Repeating a stimulus across trials led to a retrieval of the correct response even after errors, that is, even if the required response had actually not been executed. These findings suggest that episodic binding connects stimuli with intended action goals, rather than with actually performed movements. This previous work, however, pitted both types of binding and retrieval against each other so that only the stronger contribution to performance would be visible in the results. Our study was conducted to test an alternative explanation of the previous findings, which predicts the independent retrieval of both, the previously intended but not executed (i.e. correct) response, as well as the erroneously executed response, following erroneous primes, when faced with the same distractor in the probe trial. This independent retrieval could either take place in different trials or even simultaneously within one single episode. We disentangled the alternative explanations by using an experimental design with three response options (instead of two), which allowed us to conduct separate and independent tests for retrieval of goal-based bindings and bindings based on a co-activation of stimuli and erroneous responses.

The results of our study replicate previous findings in that they provide strong support for a retrieval of goal-based bindings: Repeating (vs. changing) the prime distractor in the probe led to facilitation effects if the correct prime response was also required in the probe, compared to a baseline sequence in which the prime and probe responses were unrelated. However, no evidence was found for a retrieval of bindings based on co-activation: Repeating (vs. changing) the prime distractor in the probe did not produce facilitation effects if the erroneous prime response was repeated in the probe, again compared to a baseline sequence in which the prime and probe responses were unrelated.

Our design had the advantage that it allowed for a separate, “pure” test of the two types of retrieval effects. The findings thus provide even stronger arguments against the binding of stimuli and responses on the basis of a simple co-activation mechanism. Instead, our findings add to the previous evidence for rule-based episodic storage, in which intended or “corrected” actions enter into bindings rather than actual actions (Foerster, Moeller, et al. 2021; Foerster, Rothermund, et al., 2021; Giesen et al., 2017).

The lack of support for binding and retrieval of errors based on co-activation also sheds light on the interpretation of standard SRBR effects for correct responses, as they were also found in the present study. For correct responses, actual and intended actions are identical, so that we cannot unambiguously disentangle the influence of these two types of bindings on retrieval effects empirically. The fact that we repeatedly failed to find evidence for binding and retrieval based on co-activation for errors might suggest that also for correct responses it is probably not the actual response itself that is stored and retrieved in an episodic event file. Instead, binding might target the intended action, that is, the action that was required by the target feature of the task.

However, for erroneous action episodes, coactivation-based binding between the executed erroneous response and the effect it produces emerged (Exp. 2 of Foerster, Moeller, et al., 2021). Moreover, weaker goal-based SRBR effects of relevant stimuli emerged for erroneous than correct action episodes, which might point to binding of execution-based features that only exist for executed but not for predicted correct responses (for a thorough discussion of this aspect, see Foerster et al., 2021).

The design of the present study cannot dissociate between distractor-target and distractor-response binding because target repetitions (changes) co-occurred with response repetitions (changes). However, two findings support the interpretation that the observed effects indeed reflect direct goal-based binding and retrieval of responses from distractors. For one, sequences of targets and responses have been successfully dissociated by instructing how targets map to responses in each trial and the results indicated independent processes for distractor-target, and distractor-response binding (Giesen & Rothermund, 2014). Further, we have demonstrated effects of goal-based binding and retrieval between distractors and intended correct responses after errors even if targets always changed in the analysed sequences (Foerster, Rothermund, et al., 2021). Future studies might map each of the three responses to multiple stimuli and assess distractor-response bindings after errors only for trial sequences where the target changed.

Together with the ubiquitous anecdotal evidence highlighting that actual erroneous responses that are mistakenly executed in a certain situation have an increased chance to re-occur and become chronic when the situation repeats, these findings lead us to a somewhat cautious conclusion, which is that our findings should not be taken to completely rule out the possibility of episodic binding and retrieval based on co-activation. On the one hand, although the statistical power of our experiment to detect retrieval effects of moderate size (*d_z_* = .5) was strong (1–ß > .95), a sensitivity analysis revealed that for effect sizes smaller than *d_z_* = .32, the power to detect these effects was below .8. Evidence for the null hypothesis was also anecdotal to moderate for the assessment of coactivation-based binding and retrieval effects. Still, the current results clearly demonstrate that if there are coactivation-based SRBR effects, these are likely small and less powerful than their goal-based binding counterpart, at least for the investigated situations where participants were able to handle the task relatively successfully.

However, we should also consider the possibility that episodic binding and retrieval based on co-activation did not influence responding in our experiment, but might occur under different conditions, and for different types of relations, especially since coactivation-based binding between the executed erroneous response and the effect it produces has been previously observed (Exp. 2 of Foerster, Moeller, et al., 2021). For instance, direct bindings between stimuli and errors might still occur when people become so confused that they lose track of instructions, and are no longer able to detect and correct their errors.

It seems quite likely that most errors have been registered by the cognitive system and that participants even became aware of most of their errors in our study. We found evidence for post-error slowing, with responses being slower in the probe after an erroneous (M = 388 ms) compared to a correct prime response (M = 380 ms), *t*(61) = 3.85, *p* <.001, *d_z_* = 0.49, which has been reported to indicate error awareness (Chang et al., 2014; King et al., 2010). Although SRBR effects were stronger after correct than after erroneous prime trials, which might indicate a mix of goal-based and coactivation-based binding processes in the latter condition, we consider such an explanation unlikely for the following reason: if being unaware of an error would actually lead to a binding between the distractor and the erroneous response in some of the trials, then this should be reflected in different retrieval effects for the conditions in which either the unrelated or the erroneous response is repeated in the probe (this is the major rationale of our study design). Since we did not see such a difference in retrieval effects between these two conditions, it seem unlikely that a coactivation-based binding of the erroneous response (e.g., due to unawareness) occurred in a substantial number of trials.

In addition, episodic bindings between responses and their effects (action effect learning, Foerster, Moeller, et al., 2021) might have a different logic due to a reversed temporal order in which the environmental event and response occur. Importantly, episodic binding and retrieval of response-response sequences (action sequence learning; Moeller & Frings, 2019a, b) might also be a candidate for binding based on co-activation, since this kind of response sequence learning is more incidental rather than rule-based.

In sum, it remains possible that episodic binding and retrieval based on a mere co-activation of (erroneous or correct) responses can occur under certain conditions or processing requirements. Per default, however, the cognitive system rather relies on intended or corrected actions when storing SR episodes in memory. This functional feature already shields action regulation and behavioural automatization against the intrusion of incidental errors, at least as long as people have a clear understanding of what is correct and what is not.

## Data Accessibility Statement

We are grateful to Nils Meier from our lab, for his assistance in programming the experiment that was used for this study. The preregistration (osf.io/a6yuw), analysis syntax, and data for this research (osf.io/m2btc/files) are publicly available.

## Footnotes

Also referred to as “distractor response binding and retrieval” in studies that focus on the retrieval of bindings between responses and distractors (Frings & Moeller, 2010; Giesen et al., 2012; Giesen & Rothermund, 2014; Moeller et al., 2016). In our experiment, we investigate the processes underlying stimulus response binding and retrieval by using distractors as stimuli instead of targets, since targets are typically perfectly confounded with responses. Thus, the stimulus here is irrelevant and does not predict either the target or the response.

Since error rates are not always normally distributed, we also ran arcsine transformations on this dependent variable. The pattern of results replicated for probes with correct as well as erroneous prime responses, including the main effects, and non-significant two-way interaction after erroneous primes.

We checked our results for potential variability inherent in current software implementations of Bayesian ANOVA (Pfister, 2021). The reported Bayes Factors did not represent outliers in any direction in repeated iterations of the analysis.

## References

[1] Chang, A., Chen, C.-C., Li, H.-H., & Li, C.-S. R. (2014). Event-related potentials for post-error and post-conflict Slowing. PLoS ONE, 9(6), e99909. DOI: 10.1371/journal.pone.0099909 24932780 PMC4059667

[2] Dell, G. S., Kelley, A. C., Bian, Y., & Holmes, E. W. (2019). Tuning the blueprint: How studies of implicit learning during speaking reveal the information processing components of the production system. Language, Cognition and Neuroscience, 34(9), 1246–1256. DOI: 10.1080/23273798.2019.1613553

[3] Faul, F., Erdfelder, E., Lang, A.-G., & Buchner, A. (2007). G*Power 3: A flexible statistical power analysis program for the social, behavioral, and biomedical sciences. Behavior Research Methods, 39(2), 175–191. DOI: 10.3758/BF03193146 17695343

[4] Foerster, A., Moeller, B., Huffman, G., Kunde, W., Frings, C., & Pfister, R. (2021). The human cognitive system corrects traces of error commission on the fly. Journal of Experimental Psychology: General. DOI: 10.1037/xge0001139 34807707

[5] Foerster, A., Rothermund, K., Parmar, J. J., Moeller, B., Frings, C., & Pfister, R. (2021). Goal-based binding of irrelevant stimulus features for action slips. Experimental Psychology, 68(4), 206–213. DOI: 10.1027/1618-3169/a000525 34918539 PMC8691204

[6] Frings, C., Hommel, B., Koch, I., Rothermund, K., Dignath, D., Giesen, C., Kiesel, A., Kunde, W., Mayr, S., Moeller, B., Möller, M., Pfister, R., & Philipp, A. (2020). Binding and Retrieval in Action Control (BRAC). Trends in Cognitive Sciences, 24(5), 375–387. DOI: 10.1016/j.tics.2020.02.004 32298623

[7] Frings, C., & Moeller, B. (2010). Binding targets’ responses to distractors’ locations: Distractor response bindings in a location-priming task. Attention, Perception & Psychophysics, 72(8), 2176–2183. DOI: 10.3758/APP.72.8.2176 21097861

[8] Frings, C., Rothermund, K., & Wentura, D. (2007). Distractor repetitions retrieve previous responses to targets. Quarterly Journal of Experimental Psychology, 60(10), 1367–1377. DOI: 10.1080/17470210600955645 17853245

[9] Giesen, C., Frings, C., & Rothermund, K. (2012). Differences in the strength of distractor inhibition do not affect distractor–response bindings. Memory & Cognition, 40, 373–387. DOI: 10.3758/s13421-011-0157-1 22081277

[10] Giesen, C., & Rothermund, K. (2014). Distractor repetitions retrieve previous responses and previous targets: Experimental dissociations of distractor–response and distractor–target bindings. Journal of Experimental Psychology: Learning, Memory, and Cognition, 40(3), 645–659. DOI: 10.1037/a0035278 24294915

[11] Giesen, C., Scherdin, K., & Rothermund, K. (2017). Flexible goal imitation: Vicarious feedback influences stimulus-response binding by observation. Learning & Behavior, 45(2), 147–156. DOI: 10.3758/s13420-016-0250-1 27800568

[12] Giesen, C. G., Schmidt, J. R., & Rothermund, K. (2020). The Law of Recency: An episodic stimulus-response retrieval account of habit acquisition. Frontiers in Psychology, 10, 2927. DOI: 10.3389/fpsyg.2019.02927 32010017 PMC6974578

[13] Hommel, B. (2005). How much attention does an event file need? Journal of Experimental Psychology: Human Perception and Performance, 31(5), 1067–1082. DOI: 10.1037/0096-1523.31.5.1067 16262499

[14] Hommel, B., Müsseler, J., Aschersleben, G., & Prinz, W. (2001). The Theory of Event Coding (TEC): A framework for perception and action planning. Behavioral and Brain Sciences, 24(5), 849–878. DOI: 10.1017/S0140525X01000103 12239891

[15] King, J. A., Korb, F. M., von Cramon, D. Y., & Ullsperger, M. (2010). Post-error behavioral adjustments are facilitated by activation and suppression of task-relevant and task-irrelevant information processing. Journal of Neuroscience, 30(38), 12759–12769. DOI: 10.1523/JNEUROSCI.3274-10.2010 20861380 PMC6633589

[16] Moeller, B., & Frings, C. (2019a). From simple to complex actions: Response–response bindings as a new approach to action sequences. Journal of Experimental Psychology: General, 148(1), 174–183. DOI: 10.1037/xge0000483 30211579

[17] Moeller, B., & Frings, C. (2019b). Response–response binding across effector-set switches. Psychonomic Bulletin & Review, 26(6), 1974–1979. DOI: 10.3758/s13423-019-01669-8 31654376

[18] Moeller, B., Frings, C., & Pfister, R. (2016). The structure of distractor-response bindings: Conditions for configural and elemental integration. Journal of Experimental Psychology: Human Perception and Performance, 42(4), 464–479. DOI: 10.1037/xhp0000158 26501840

[19] Pfister, R. (2021). Variability of Bayes Factor estimates in Bayesian analysis of variance. The Quantitative Methods for Psychology, 17(1), 40–45. DOI: 10.20982/tqmp.17.1.p042

[20] Rothermund, K., Wentura, D., & De Houwer, J. (2005). Retrieval of incidental stimulus-response associations as a source of negative priming. Journal of Experimental Psychology: Learning, Memory, and Cognition, 31(3), 482–495. DOI: 10.1037/0278-7393.31.3.482 15910132

[21] Smalle, E. H. M., Muylle, M., Duyck, W., & Szmalec, A. (2021). Less is more: Depleting cognitive resources enhances language learning abilities in adults. Journal of Experimental Psychology: General. DOI: 10.1037/xge0001058 33856850

[22] Wood, W., & Rünger, D. (2016). Psychology of habit. Annual Review of Psychology, 67(1), 289–314. DOI: 10.1146/annurev-psych-122414-033417 26361052

